# Clinical determinants of early spontaneous conversion to sinus rhythm in patients with atrial fibrillation

**DOI:** 10.1007/s12471-020-01528-5

**Published:** 2021-01-06

**Authors:** N. A. H. A. Pluymaekers, E. A. M. P. Dudink, B. Weijs, K. Vernooy, D. E. J. Hartgerink, J. S. Jacobs, Ö. Erküner, N. G. H. M. Marcks, Y. J. M. van Cauteren, T. Dinh, R. M. A. ter Bekke, J. E. M. W. Sels, T. S. R. Delnoij, Z. Geyik, R. G. H. Driessen, D. K. Linz, D. W. den Uijl, H. J. G. M. Crijns, J. G. L. M. Luermans

**Affiliations:** 1 Department of Cardiology and Cardiovascular Research Institute Maastricht (CARIM), Maastricht University Medical Centre (MUMC+), Maastricht, The Netherlands; 2 Department of Intensive Care Medicine, Maastricht University Medical Centre (MUMC+), Maastricht, The Netherlands; 3 Department of Cardiology, Radboud University Medical Centre, Nijmegen, The Netherlands

**Keywords:** Acute atrial fibrillation, Spontaneous conversion, Determinants, Treatment, Cardioversion, Wait-and-see approach

## Abstract

**Background:**

The current standard of care for acute atrial fibrillation (AF) focuses primarily on immediate restoration of sinus rhythm by cardioversion, although AF often terminates spontaneously.

**Objective:**

To identify determinants of early spontaneous conversion (SCV) in patients presenting at the emergency department (ED) because of AF.

**Methods:**

An observational study was performed of patients who visited the ED with documented AF between July 2014 and December 2016. The clinical characteristics and demographics of patients with and without SCV were compared.

**Results:**

We enrolled 943 patients (age 69 ± 12 years, 47% female). SCV occurred within 3 h of presentation in 158 patients (16.8%). Logistic regression analysis showed that duration of AF <24 h [odds ratio (OR) 7.7, 95% confidence interval (CI) 3.5–17.2, *p* < 0.001], left atrial volume index <42 ml/m^2^ (OR 1.8, 95% CI 1.2–2.8, *p* = 0.010), symptoms of near-collapse at presentation (OR 2.4, 95% CI 1.2–5.1, *p* = 0.018), a lower body mass index (BMI) (OR 0.9, 95% CI 0.91–0.99, *p* = 0.028), a longer QTc time during AF (OR 1.01, 95% CI 1.0–1.02, *p* = 0.002) and first-detected AF (OR 2.5, 95% CI 1.6–3.9, *p* < 0.001) were independent determinants of early SCV.

**Conclusion:**

Early spontaneous conversion of acute AF occurs in almost one-sixth of admitted patients during a short initial observation in the ED. Spontaneous conversion is most likely to occur in patients with first-onset, short-duration AF episodes, lower BMI, and normal left atrial size.

## What’s new?

- Spontaneous conversion is most likely to occur in patients with first-onset, short-duration episodes of atrial fibrillation (<24 h), lower body mass index, and normal left atrial size.
- The results of the current study facilitate the identification of patients with a high likelihood of spontaneous conversion to sinus rhythm and the implementation of a wait-and-see approach.

## Introduction

Atrial fibrillation (AF) is a commonly encountered arrhythmia and causes a significant health care burden [1, 2]. The prevalence of AF, and thereby the economic costs (mainly for hospitalisation and treatment), continues to increase [3, 4]. Current treatment is focused primarily on appropriate anticoagulation, rate or rhythm control strategy, and the assessment of underlying conditions that predispose to AF. In patients with acute symptomatic AF, the primary aim of treatment is early restoration of sinus rhythm (SR) by pharmacological cardioversion (PCV), electrical cardioversion (ECV) or a combination of both [4]. However, it could be questioned whether immediate restoration by means of cardioversion is necessary, since several previous studies have reported that spontaneous conversion (SCV) of AF to SR occurs in up to 70% of acute AF cases [1, 5–9], making prompt ECV or PCV unnecessary. Appropriate identification of patients with a high likelihood of SCV of AF is needed. The aim of this study is to determine the clinical characteristics associated with early SCV in patients presenting with AF at the emergency department (ED).

## Methods

### Setting

We conducted an observational study of 943 adult patients who visited the ED with AF between July 2014 and December 2016. Patients were included at the Maastricht University Medical Centre+ (MUMC+). The study was approved by the Institutional Review Board of the MUMC+.

### Study population

All patients were aged over 18 years. The patients either had electrocardiographic documentation of AF at presentation or were patients with pre-hospital conversion if they had previous electrocardiographically documented AF with a verified symptom-rhythm correlation or if the current episode was electrocardiographically documented by the general practitioner and patients converted on their way to the hospital. For the purpose of this study the following exclusion criteria were applied: a history of persistent or permanent AF, haemodynamic instability, or signs of acute coronary syndrome or heart failure at initial work-up. Patients were treated at the discretion of the treating physician; no additional study-related action was taken. The institutional protocol allowed for both forms of cardioversion, depending on patients’ profiles, timing of eventual cardioversion in relation to last meal, and physicians’ preferences and experiences.

### Data collection

Baseline characteristics were collected, including age, sex, duration of AF, current symptoms, medical history, and (prior) echocardiographic data. Patients underwent a full physical examination, a 12-lead electrocardiogram analysed with software from the MUSE system (GE Medical Systems, Milwaukee, WI, USA), and laboratory investigation at the ED. Information regarding therapeutic strategy including rate versus rhythm control was noted. We compared characteristics between patients with SCV and non-spontaneous conversion (non-SCV).

### Definitions

For the purpose of this study patients with a history of persistent or permanent AF, defined as a previous episode of AF lasting longer than >48 h, were excluded. The duration of the current episode was not an exclusion criterion; therefore episodes longer than 48 h were also included if the patient had no history of persistent or permanent AF. Conversion was defined as spontaneous if the patient converted to SR without active cardioversion, either ECV or PCV, before presentation at the ED or within 3 h after ED presentation, which was the time interval allowing proper work-up towards active cardioversion and during which spontaneous cardioversion could happen.

### Statistical analysis

Data management and analysis were performed using IBM SPSS version 25 (Armonk, NY, USA). Results were reported as mean ± standard deviation or median with interquartile range (IQR). A chi-square or Fisher’s exact test was used to compare categorical variables. Normally distributed continuous covariates were compared using the Student’s *t*-test. For comparison of skewed continuous covariates the Mann-Whitney U test was used. A binary logistic regression was performed, using backward selection until all variables in the model reached a *p*-value <0.05, to identify possible determinants for SCV. All variables showing a significant (*p* < 0.05) univariable relationship for SCV were included in the regression analysis. A *p*-value of <0.05 was considered statistically significant.

## Results

**Fig. 1:**
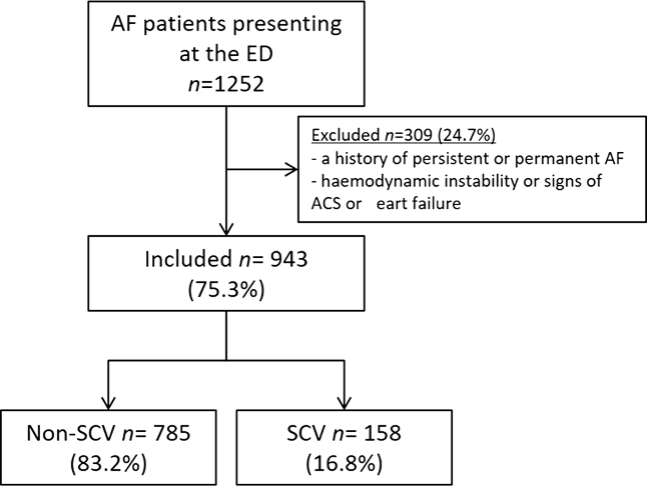
Study flow chart. Exclusion criteria were a history of persistent or permanent atrial fibrillation (*AF*), haemodynamic instability, or signs of acute coronary syndrome (*ACS*) or heart failure at presentation. Persistent AF was defined for this trial as a previous episode lasting longer than 48 h. haemodynamic instability was defined as a heart rate above 170 bpm or a systolic blood pressure below 100 mm Hg. *ED* emergency department, *SCV* spontaneous conversion

We enrolled 943 consecutive patients (Fig. 1) with a mean age of 69 ± 12 years, 47% females. Expressed symptoms at presentation were palpitations in 74% of patients, dyspnoea in 28%, chest pain in 20%, dizziness in 16%, fatigue in 8%, near-collapse in 7%, and 10% experienced other symptoms of AF. An overview of the baseline characteristics of SCV and non-SCV patients is presented in Tab. 1.

**Table 1:** Baseline characteristics according to spontaneous cardioversion

Total, *n* = 943	Non-SCV *n* = 785 (83.2%)		SCV *n* = 158 (16.8%)		*p*-value (non-SCV vs SCV)
*Demographics*					
Age, years (±SD)	68.5	(±12.6)	69.7	(±11.3)	0.257
Female, *n* (%)	361	(46.0%)	85	(53.8%)	0.073
BMI, kg/m^2^ (±SD)	28.5	(±6.5)	27.0	(±4.6)	0.001
*History*					
Hypertension, *n* (%)	475	(60.5%)	107	(67.7%)	0.089
Diabetes mellitus, *n* (%)	101	(12.9%)	23	(14.6%)	0.566
Hypercholesterolaemia, *n* (%)	285	(36.3%)	41	(25.9%)	0.013
Smoking:					
– Current, *n* (%)	87	(11.1%)	16	(10.1%)	0.725
– Past, *n* (%)	69	(8.8%)	12	(7.6%)	0.625
Myocardial infarction, *n* (%)	90	(11.5%)	18	(11.4%)	0.979
Coronary artery disease, *n* (%)	140	(17.8%)	27	(17.1%)	0.823
Percutaneous coronary intervention, *n* (%)	81	(10.3%)	16	(10.2%)	0.962
Coronary artery bypass graft, *n* (%)	59	(7.5%)	3	(1.9%)	0.009
Valve surgery, *n* (%)	42	(5.6%)	4	(1.3%)	0.021
Stroke					
– Ischaemic, *n* (%)	36	(4.6%)	3	(1.9%)	0.122
– Haemorrhagic, *n* (%)	0	(0%)	1	(0.6%)	0.168
Transient ischaemic attack, *n* (%)	31	(3.9%)	9	(5.7%)	0.320
Pulmonary embolism, *n* (%)	8	(1.0%)	1	(0.6%)	>0.999
Deep venous thrombosis, *n* (%)	2	(0.2%)	3	(1.9%)	0.036
Congenital heart disease, *n* (%)	10	(1.3%)	2	(1.3%)	>0.999
Hyperthyroidism, *n* (%)	18	(2.3%)	7	(4.4%)	0.168
Hypothyroidism, *n* (%)	19	(2.4%)	5	(3.2%)	0.588
Chronic obstructive pulmonary disease, *n* (%)	30	(3.8%)	1	(0.6%)	0.040
Peripheral artery disease, *n* (%)	26	(3.3%)	7	(4.4%)	0.485
Obstructive sleep apnoea syndrome, *n* (%)	18	(2.3%)	5	(3.2%)	0.569
Atrial flutter, *n* (%)	89	(11.3%)	23	(14.6%)	0.254
ICD, *n* (%)	29	(3.7%)	2	(1.2%)	0.118
PM, *n* (%)	20	(2.5%)	4	(2.5%)	>0.999
Ablation therapy for AF, *n* (%)	87	(11.1%)	12	(7.6%)	0.192
CHA_2_DS_2_-VASc (±SD)	2.6	(±1.6)	2.7	(±1.5)	0.370
*Medication at baseline*					
Vitamin K antagonist, *n* (%)	281	(35.8%)	41	(25.9%)	0.017
Direct oral coagulants, *n* (%)	144	(18.3%)	32	(20.3%)	0.574
***Other medication***					
Acetylsalicylic acid, *n* (%)	88	(11.2%)	23	(14.6%)	0.234
ACE inhibitors, *n* (%)	142	(18.1%)	27	(17.1%)	0.765
ARB, *n* (%)	246	(31.3%)	50	(31.8%)	0.900
Spironolactone, *n* (%)	19	(2.4%)	2	(1.3%)	0.556
Beta-blocker, *n* (%)	353	(45.0%)	68	(43.0%)	0.656
Digoxin, *n* (%)	37	(4.7%)	5	(3.2%)	0.388
AAD use, *n* (%)	231	(29.4%)	39	(24.7%)	0.229
Statin, *n* (%)	303	(38.6%)	56	(35.4%)	0.449
*Echocardiography*^**a**^					
LAV index <42 ml/m^2^, *n* (%)	317	(47.8%)	84	(61.3%)	0.004
Normal RAV index ml/m^2^, *n* (%)	258	(32.9%)	66	(41.8%)	0.031
LVH, *n* (%)	248	(31.6%)	47	(29.7%)	0.648
LVEF (±SD)	59.5	(±7.5)	61.6	(±5.0)	0.002
*AF characteristics*					
First-detected AF, *n* (%)	251	(32.0%)	66	(41.8%)	0.017
Duration of symptoms <24 h, *n* (%)	534	(68.0%)	143	(90.5%)	<0.001
Mean systolic blood pressure, mm Hg (±SD)	135.0	(±20.4)	134.2	(±26.2)	0.735
Mean diastolic blood pressure, mm Hg (±SD)	85.0	(±15.1)	82.2	(±14.9)	0.044
Palpitations, *n* (%)	565	(72.0%)	129	(81.6%)	0.012
Dyspnoea, *n* (%)	232	(29.6%)	28	(17.7%)	0.002
Fatigue, *n* (%)	72	(9.2%)	5	(3.2%)	0.064
Near-collapse, *n* (%)	48	(6.1%)	18	(11.4%)	0.018
Chest pain, *n* (%)	146	(18.6%)	44	(27.8%)	0.008
Dizziness, *n* (%)	117	(14.9%)	32	(20.3%)	0.093
Other symptoms of AF, *n* (%)	79	(10.1%)	16	(10.1%)	0.981
*ECG*					
Heart rate, bpm (±SD)	118	(±27)	125	(±29)	0.004
QRS duration, ms (±SD)	94	(±21)	96	(±25)	0.356
LBBB, *n* (%)	21	(2.7%)	6	(4.7%)	0.260
QTc, ms (±SD)	452	(±34)	460	(±34)	0.035
*Laboratory results*					
Potassium, mmol/l (*n* = 877) (±SD)	4.2	(±0.5)	4.2	(±0.6)	0.277
Creatinine, umol/l (*n* = 578) (±SD)	95.3	(±42.7)	104.7	(±103.4)	0.142
Haemoglobin, mmol/l (*n* = 423) (±SD)	8.6	(±1.1)	8.4	(±1.2)	0.094
TSH, mU/l (*n* = 516) (±SD)	2.6	(±5.9)	2.9	(±3.6)	0.602

The echocardiographic data were from 28 months before presentation to 74 months after (mean interval −3 weeks)

*AAD* antiarrhythmic drug, *ACE* angiotensin-converting enzyme, *AF* atrial fibrillation, *ARB* angiotensin receptor blockers, *BMI* body mass index, *CI* confidence interval, *ECG* electrocardiography, *ICD* implantable cardioverter-defibrillator, *LAV* left atrial volume, *LBBB* left bundle branch block, *LVEF* left ventricular ejection fraction, *LVH* left ventricular hypertrophy (>9 mm), *PM* pacemaker, *RAV* right atrial volume, *SCV* spontaneous conversion, *TSH* thyroid-stimulating hormone

^a^ Based on *n* = 854 due to missing data. Normal RAV index for men 25 ± 7 ml/m^2^, women 21 ± 6 ml/m^2^

### Cardioversion

**Fig. 2:**
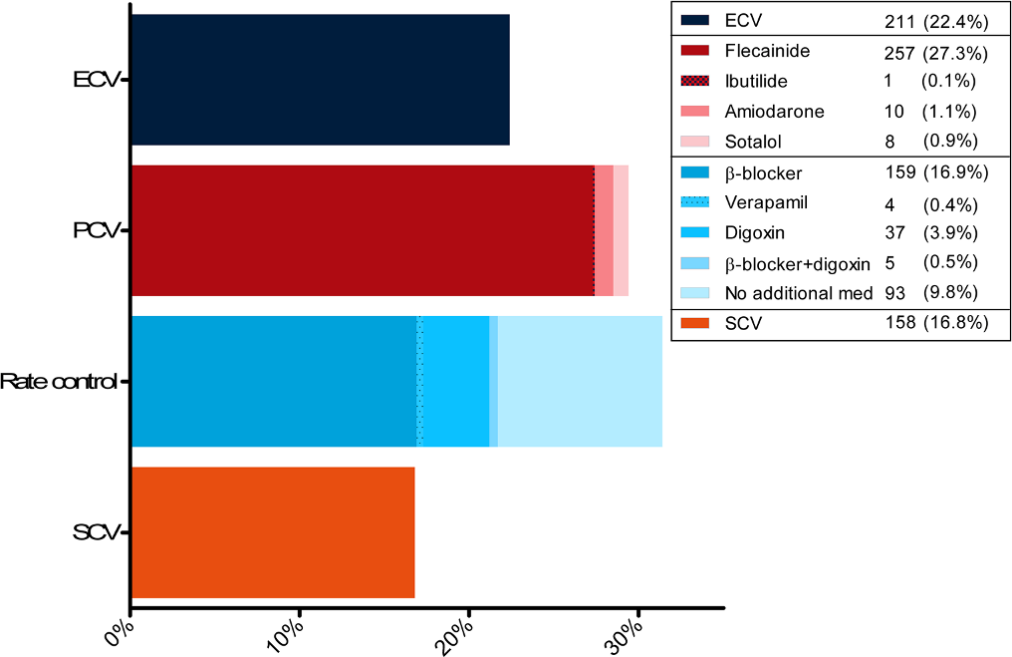
Overall treatment strategy. *ECV* electrical cardioversion, *PCV* pharmacological cardioversion, *SCV* spontaneous conversion

Spontaneous conversion to SR occurred in 158 (16.8%) patients. In patients without SCV, an active cardioversion was attempted in 487 (51.6%), rate control was chosen in 31.4%, and 2 patients received a pacemaker (0.2%) because of sick sinus syndrome. Pharmacological cardioversion was performed in 276 of 487 (56.7%) patients; flecainide was the preferred drug in the majority of cases (93.1%). Electrical cardioversion was performed in 211 of 487 (43.3%) patients. Pharmacological cardioversion was successful in 80.1% of cases, ECV in 92.9%; the overall success rate of cardioversion was 85.8%. An overview of the treatment strategy is presented in Fig. 2.

### Spontaneous conversion

The median duration from onset of symptoms until the end of the observation period was 4.0 h (IQR 7, range 0–86 h) in patients with SCV, and 11.0 h (IQR 19, range 3–1355 h) in patients without SCV. The median observation time at the ED pending SCV was 32 min (IQR 81, range 0–165 min). The number of patients in SR after 1 h was 100 (10.6%), and after 2 h 138 (14.6%). The mean age of the SCV and non-SCV groups was comparable (70 ± 11 vs 69 ± 13 years, *p* = 0.257, respectively). There was a trend towards more spontaneous cardioversions in female patients (53.8 vs 46.0%, *p* = 0.073). Patients with SCV less frequently had a history of hypercholesterolaemia (25.9 vs 36.3%, *p* = 0.013), coronary artery bypass grafting (1.9 vs 7.5%, *p* = 0.009), chronic obstructive pulmonary disease (0.6 vs 3.8%, *p* = 0.040), and less frequently had a history of valve surgery (1.3 vs 5.6%, *p* = 0.021). Mean body mass index (BMI) was lower in patients with SCV (27.0 vs 28.5 kg/m^2^, *p* = 0.001). First-detected AF was more common in patients with SCV than in non-SCV patients (41.8 vs 32.0%, *p* = 0.017, respectively). In addition, patients with SCV had a shorter median duration of symptoms (3.0 h; IQR 5 vs 8.0; IQR 19, *p* < 0.001) and more often suffered symptoms of near-collapse (11.4 vs 6.1%, *p* = 0.018), palpitations (81.6 vs 72.0%, *p* = 0.012), chest pain (27.8 vs 18.6%, *p* = 0.008), and dizziness (20.3 vs 14.9%, *p* = 0.093). Patients complaining of near-collapse more often had first-detected AF (58% vs 42%, *p* < 0.001). Patients with SCV more often had a higher ventricular rate (125 vs 118 bpm, *p* = 0.004), longer QTc time during AF (460 ms ± 34 vs 452 ms ± 34, *p* = 0.035) and more often had a lower left atrial volume index (LAVI) (<42 ml/m^2^) (61.3 vs 47.8%, *p* = 0.004). A longer QTc was observed in patients who had a breakthrough arrhythmia (460 ± 40 ms in patients with antiarrhythmic drugs (AAD) vs 450 ± 40 ms in patients without AAD, *p* < 0.001).

Logistic regression analysis showed that duration of AF <24 h (OR 7.7, 95% CI 3.5–17.2, *p* < 0.001), LAVI <42 ml/m^2^ (OR 1.8, 95% CI 1.2–2.8, *p* = 0.010), symptoms of near-collapse at presentation (OR 2.4, 95% CI 1.2–5.1, *p* = 0.018), a lower BMI (OR 0.9, 95% CI 0.91–0.99, *p* = 0.028), a longer QTc time during AF (OR 1.01, 95% CI 1.0–1.02, *p* = 0.002) and first-detected AF (OR 2.5, 95% CI 1.6–3.9, *p* < 0.001) were independent determinants of early SCV (Tab. 2).

**Table 2:** Univariable and multivariable regression analyses for predictors of spontaneous conversion to sinus rhythm. This model is based on *n* = 759 due to missing data on left atrial volume index

	Univariable					Multivariable		
	Non-SCV, *n* = 785		SCV, *n* = 158		*p*-value	OR	95% CI	*p*-value
*Demographics*								
BMI	28.5	(±6.5)	27.0	(±4.6)	0.001	0.9	0.91–0.99	0.028
*Presentation characteristics*								
Duration <24 h	534	(68.0%)	143	(90.5%)	<0.001	7.7	3.5–17.2	<0.001
First-detected AF	251	(32.0%)	66	(41.8%)	0.017	2.5	1.6–3.9	<0.001
Near-collapse	48	(6.1%)	18	(11.4%)	0.018	2.4	1.2–5.1	0.018
*Echo- and electrocardiography*								
QTc (ms)	452	(±34)	460	(±34)	0.035	1.01	1.00–1.02	0.002
LAV index <42 ml/m^2^	317	(47.8%)	84	(61.3%)	0.004	1.8	1.2–2.8	0.010

*AF* atrial fibrillation, *BMI* body mass index, *CI* confidence interval, *LA* left atrial, *OR* odds ratio, *SCV* spontaneous conversion

In patients with a duration of AF <24 h, first-detected AF, and smaller LAVI, the SCV rate was high: 38% (38 of 99 patients).

## Discussion

The present study showed that early SCV to SR occurred in 16.8% of patients presenting with AF at the ED within a median time at the ED of 32 min. Duration of symptoms <24 h, first-detected AF, LAVI <42 ml/m^2^, a lower BMI and symptoms of near-collapse or longer QTc time at presentation were independent determinants of early SCV.

Previous studies have reported variable SCV rates of acute AF ranging from 26 to 71% [1, 5–11], mostly depending on differences in study design, observation period and patient selection criteria. Danias et al. included 356 in-hospital patients with symptomatic AF <72 h and observed a SCV rate of 68%. In the absence of initial AAD therapy, these investigators found that the best predictor of SCV was a duration of symptoms <24 h [1]. This was confirmed by Lindberg et al. [6], who reported a SCV rate of 54% in patients in hospital with first-onset AF (*n* = 374). In 153 patients with paroxysmal AF and a symptom duration <24 h and without structural heart disease, heart failure or hyperthyroidism, Geleris and co-workers observed a SCV rate of 71.2%. In this homogeneous group, small left atrial size was the only predictor for SCV [7]. Boriani et al. performed a randomised controlled trial of propafenone versus placebo for recent-onset AF (<7 days) and observed a SCV rate in the placebo group of 37% after 8 h of observation [11]. Sub-analyses of this study showed a higher likelihood of SCV in patients without underlying heart disease [11] and those aged <60 years[12].

The lower rate of SCV observed in our cohort is likely the result of shorter observation periods (censored at 3 h maximum) at the ED. The SCV rate we found is comparable to that in other reports of recent-onset AF by Vinson et al. (28.6%) [13] and Stiell et al. (26.5%) [10]. Vinson et al. investigated the management of recent-onset AF or atrial flutter at the ED. A total of 206 patients were included with a duration of symptoms <48 h. They found a SCV rate of 28.6% [13]. A similar SCV rate was reported by Stiell et al., who included 1068 patients with recent-onset AF (<48 h) at the ED. During a median observation period of 6.7 h, 26.5% of patients had SCV [10]. Sub-analyses of the ENSURE-AF trial, which included patients with non-valvular atrial fibrillation with a duration >48 h and who were scheduled for ECV on anticoagulation therapy, have demonstrated SCV rates of 7.6%. In the sub-analysis of Cohen et al., a history of paroxysmal AF was the only predictor for SCV [14].

To the best of our knowledge, our study is the largest concerning determinants of early SCV and enables clinicians to identify patients who are most likely to convert spontaneously to SR. In our cohort, the following characteristics appeared to be determinants of early SCV at the ED: AF duration <24 h, first-detected AF, a lower LAVI, a lower BMI, a longer QTc time during AF and symptoms of near-collapse at presentation. These parameters are most likely indicative of early phases of AF evolution. A lower LAVI suggests that macroscopic electrical and structural remodelling of the atrium has not been evident yet or is at an early stage. It is well known that this is related to higher conversion rates [15, 16]. Obesity is a well-known risk factor for developing AF [17] and a higher BMI is independently associated with progression from paroxysmal to permanent AF [18]; this could explain the higher SCV rate in patients with a lower BMI. Patients presenting with near-collapse during AF more often had first-detected AF, which may be related to the higher SCV rates seen in those patients.

### ‘Wait-and-see approach’ in acute AF

The substantial rates of SCV of acute AF in this and other reports [1, 5–8, 10], combined with the rising prevalence of AF, cost aspects and the possible complications associated with acute cardioversion may justify less aggressive arrhythmia management. A wait-and-see strategy for patients with stable AF with onset of symptoms <48 h encompasses standard rate control measures, adequate initiation of anticoagulation and delayed cardioversion within 48 h if necessary. In a small study investigating this wait-and-see approach, two-thirds of 35 patients had converted spontaneously within 48 h [5]. This finding was recently confirmed by the RACE 7 ACWAS trial, a multicentre randomised controlled trial which compared a wait-and-see approach (symptom alleviation and delayed cardioversion when necessary) with immediate cardioversion in patients with recent-onset AF [9, 19]. This study showed that a wait-and-see approach is non-inferior with respect to the presence of SR at 4 weeks when compared to immediate cardioversion; almost 70% of the patients in the wait-and-see group had SCV to SR within 48 h. Those results support the opinion that a wait-and-see approach is a worthy alternative to discuss with patients but will not replace early cardioversion completely. Early cardioversion could be beneficial, since it shortens the time until conversion, which might eliminate symptoms earlier. However, in the above-mentioned study symptom control was similar in both groups [9]. Another advantage of early pharmacological conversion is the observation of the antiarrhythmic response, which tests the safety of a pill-in-the-pocket approach [20]. Therefore, patients with stable recent-onset AF and their physicians might choose between the two approaches in a shared decision-making process.

The results of the current study facilitate the identification of patients with a high likelihood of SCV to SR and the implementation of a wait-and-see approach. Identification of acute AF patients susceptible to spontaneous restoration of SR may reduce the number of emergency visits (especially in stable patients with recurrent episodes and a known symptom-rhythm correlation), costs and unnecessary and potentially harmful treatment.

### Limitations

Since this study was not designed to evaluate SCV rates, our results are probably an underestimation of the true SCV rate: patients who received active cardioversion might have converted spontaneously if a wait-and-see approach had been adopted. Due to the observational study design we could not adjust for this possible bias. Patients with pre-hospital SCV were included in the analysis. Even if the likelihood of those patients having AF was extremely high, one cannot be completely certain that those patients had an actual episode of AF, which could have led to an overestimation of the SCV rate.

## Conclusion

Early SCV of acute AF occurs in almost one-sixth of admitted patients during a short initial observation in the ED. Spontaneous conversion is most likely to occur in patients with first-onset, short-duration AF episodes, lower BMI, and normal left atrial size.
